# Long-read sequencing infers a mechanism for copy number variation of template for alternative lengthening of telomeres in a wild *C. elegans* strain

**DOI:** 10.17912/micropub.biology.000563

**Published:** 2022-05-03

**Authors:** Bo Yun Lee, Jun Kim, Junho Lee

**Affiliations:** 1 Research Institute of Basic Sciences, Seoul National University, Seoul, Korea; 2 Institute of Molecular Biology and Genetics, Seoul National University, Seoul, Korea; 3 Department of Biological Sciences, Seoul National University, Seoul, Korea

## Abstract

Template for alternative lengthening of telomeres 1 (TALT1) is a specific sequence used to protect chromosomal ends from telomere damage first identified in the CB4856 strain of *Caenorhabditis elegans* . Here we assembled the genome of DL226, a wild strain with one more copy of TALT1-like sequences in its genome compared to those of CB4856, using long-read DNA sequencing. We found that a five-copy array of short telomeric repeats and TALT1s present in CB4856 were changed to a six-copy array due to the duplication of the third copy; there was an additional damage-repair trace in the new short telomeric repeat near the newly replicated TALT1.

**Figure 1. The structure of TALT1 arrays in CB4856 and DL226:**
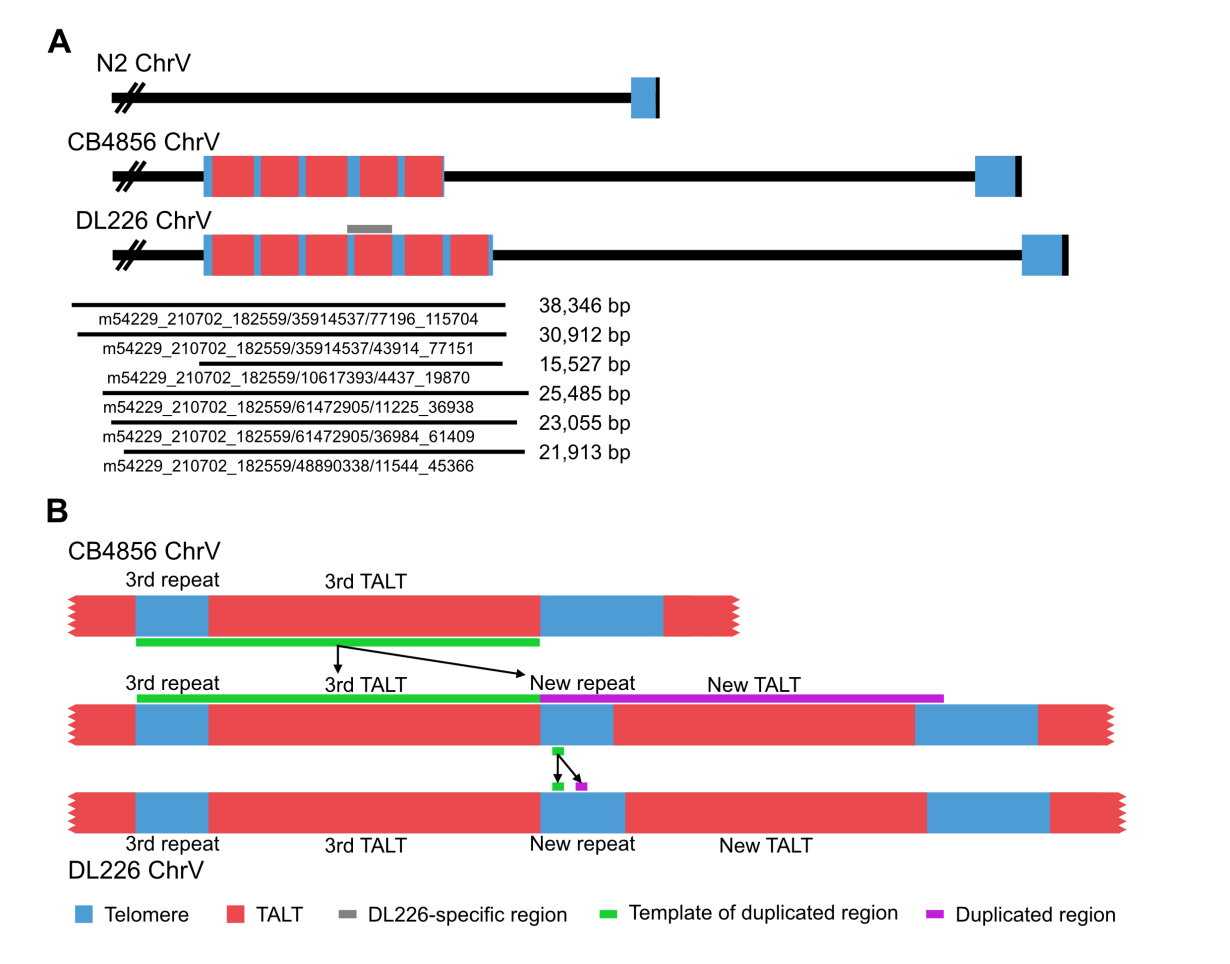
(A) Schematic representation of chromosome V right arm regions in N2, CB4856, and DL226. Locations of TALT sequences are V:21149554-21162688 in the CB4856 genome and V:21278369-21293965 in the DL226 genome. Six raw reads covered the 6-copy TALT array of DL226. DL226-specific TALT insertion was indicated by a gray horizontal line. The six raw-read sequences are available in Extended Data 2. (B) The comprehensive structure of duplicated regions. The new telomeric repeat and TALT (the long purple line) are right next to the original copy (the long green line). Besides, a duplicated part of telomeric repeat (the short purple line) is also in the repeat.

## Description

One of the *C* . *elegans* wild strains, CB4856, has a unique subtelomere structure at the right end of chromosome V compared to the N2 genome in that the CB4856 genome has five tandem copies of short telomeric repeat and TALT1 (Kim et al., 2019). It is suggested that these telomeric repeats and TALTs were duplicated through the repair process of telomere damage in the ancestor of CB4856 during evolution (Kim et al., 2019; Kim et al., 2020). In addition to the CB4856 strain, six other wild strains, which include QX1794, QX1793, DL226, CX11262, CX11315, and CX11264, exhibited similar copy-number variations for TALTs as analyzed by short-read sequencing; interestingly, DL226 and CX11315 have one and two additional copies of TALTs, respectively (Kim et al., 2019). To determine whether additional TALT copy numbers could be a trace of another telomere damage and repair, we assembled a draft genome of DL226 using long-read sequencing and analyzed the structure containing the new TALT replication.

We obtained 7.4 Gb data (52× mapping coverage; 776,415 reads; 15,654 bp of N50 length) by sequencing DL226 genomic DNA using the Pacific Biosciences (PacBio) Sequel platform (Extended data, Table 1). We assembled these reads into 79 contigs of about 103 Mb, and then the contigs were scaffolded using homology between our DL226 contigs and the N2 genome (Extended data, Table 2). To check the copy number and location of the new TALT replication, we searched the TALT sequence using BLASTn (Altschul et al., 1990; Camacho et al., 2009). As a result, we found that the right end of chromosome V in DL226 contained six copies of TALTs rather than five copies found in CB4856 (Figure 1A). Additionally, there was the new short telomeric repeat on the left of the new TALT.

We can guess where new short telomeric repeat and TALT sequences originated from because different mutations are accumulated in the existing five copies of short telomeric repeat and TALT sequences. We compared sequences of the five-copy array of CB4856 with those of the six-copy array of DL226 and found that except for the fourth copy of DL226, the remaining copies were very similar (> 97.3% Identity) with those of CB4856 in order. We also observed that the fourth copy of DL226 was almost the same as the third copy of CB4856 except for duplicated regions in the short telomeric repeat (Figure 1B). We suppose that this insertion may have occurred via two steps: First, the third telomeric repeats and TALT sequences were duplicated using homology of telomeric repeats, and then a small block of variant telomeric repeats in the new fourth copy of telomeric repeats was duplicated again.

## Methods

***C. elegans* maintenance and DNA sequencing.**

DL226 worms were cultured under standard culture conditions (Brenner, 1974; Sulston and Hodgkin, 1988). Genomic DNA of mixed-stage worms was extracted as described in Kim et al., 2019 and sequenced using the continuous long-read mode of the PacBio Sequel platform by Macrogen, Korea (https://dna.macrogen.com/).

**Genome assembly and scaffolding.**

Genome assembly was conducted with PacBio reads using Canu (version 2.2; *canu useGrid=false genomeSize=102m -pacbio DL226.fa.gz* ) (Koren el al., 2017). We used the N2 genome (WS282) as a reference genome to scaffold these 79 contigs using RagTag (version 2.0.1; ragtag.py scaffold) (Alonge et al., 2019).

**Finding TALTs and discovering the origins of the new telomeric repeat and TALT.**

To find TALT sequences in the DL226 genome, we built a BLAST database of the DL226 genome using Makeblastdb (version 2.7.1+; *makeblastdb -input_type fasta -dbtype nucl -parse_seqids* ). We then searched TALT sequence in the DL226 genome database using BLASTn (version 2.7.1+; default setting with e-value < 0.001) (Altschul et al., 1990; Camacho et al., 2009). Next, to identify which copy of the telomeric repeat and TALT in the CB4856 genome is similar with the additional telomeric repeat and TALT in the DL226 genome, we aligned the additional telomeric repeat and TALT of DL226 to the telomeric repeats and TALTs of CB4856 using Clustal Omega (Madeira et al., 2019).

**Data availability.**

Our genome assembly and raw PacBio reads of DL226 were submitted to the NCBI BioProject database (https://www.ncbi.nlm.nih.gov/bioproject) under accession number PRJNA819174.
